# Immobilization of Firefly Bioluminescent System: Development and Application of Reagents

**DOI:** 10.3390/bios13010047

**Published:** 2022-12-28

**Authors:** Elena N. Esimbekova, Maria A. Kirillova, Valentina A. Kratasyuk

**Affiliations:** 1 Institute of Fundamental Biology and Biotechnology, Siberian Federal University, 79 Svobodny Avenue, 660041 Krasnoyarsk, Russia; 2 Institute of Biophysics SB RAS, 50/50 Akademgorodok, 660036 Krasnoyarsk, Russia

**Keywords:** firefly luciferase, immobilization, microbial contamination, gelatin, bovine serum albumin, dithiothreitol

## Abstract

The present study describes the method of preparing reagents containing firefly luciferase (FLuc) and its substrate, D-luciferin, immobilized into gelatin gel separately or together. The addition of stabilizers dithiothreitol (DTT) and bovine serum albumin (BSA) to the reagent is a factor in achieving higher activity of reagents and their stability during storage. The use of immobilized reagents substantially simplifies the procedure of assay for microbial contamination. The mechanism of action of the reagents is based on the relationship between the intensity of the bioluminescent signal and the level of ATP contained in the solution of the lysed bacterial cells. The highest sensitivity to ATP is achieved by using immobilized FLuc or reagents containing separately immobilized FLuc and D-luciferase. The limit of detection of ATP by the developed reagents is 0.3 pM, which corresponds to 20,000 cells·mL^−1^. The linear response range is between 0.3 pM and 3 nM ATP. The multicomponent reagent, containing co-immobilized FLuc and D-luciferin, shows insignificantly lower sensitivity to ATP—0.6 pM. Moreover, the proposed method of producing an immobilized firefly luciferin-luciferase system holds considerable promise for the development of bioluminescent biosensors intended for the analysis of microbial contamination.

## 1. Introduction

Recent years have seen considerable advances in the development of optical biosensors, including bioluminescent ones. In bioluminescent optical biosensors, light emission is caused by chemical reactions occurring in a living organism [1]. Most of the bioluminescent biosensors used in biological and biotechnological research are based on the firefly enzyme system. For example, biosensors based on firefly luciferase (FLuc) are used to analyze gene expression, visualize disease states, and conduct high-throughput screening in drug development [2]. The FLuc-based biosensors have the widest application in the food industry to determine the degree of bacterial contamination of foodstuff and beverages and, thus, assess their quality [3]. Methods have been worked out to assess the quality of milk [4,5,6], beer [7,8,9], drinking water [10,11], and meat [12]. A somatic cell count assay has been proposed as a diagnostic indicator of cow mastitis [13]. Assays have been developed to monitor the hygiene of work surfaces used for meat and vegetable cutting in the food industry [14], in hospital catering units [15], and in milk processing plants [16]. The technical implementation of the idea of assessing microbial contamination by measuring ATP enables the use of this approach in home hygiene practices, e.g., to determine the cleanliness of hands and tables [17].

Although the method has numerous advantages, and the assay is easy and quick to perform, there are factors that limit the use of this method, such as the instability of the enzyme system during use [18], the limited storage time of assay reagents, and the necessity of creating a microenvironment for the enzyme (pH, temperature, etc.). For FLuc to remain active and capable of serving as a biorecognition element in biosensors, mutant and chimeric forms of the enzyme have been created [2,19,20,21,22,23]. The microenvironment of the enzyme can be varied to produce enzyme preparations that will both have high catalytic activity and retain stable bioluminescent signal during long-term storage. This, in turn, provides the basis for automating the assays using stabilized enzymes. Such results can be achieved by adding stabilizers [24,25], which also enhance enzyme efficiency, and by immobilizing the enzymes onto various carriers.

Immobilization techniques include binding with the carrier, encapsulation, and cross-linking [26]. Different authors reported FLuc immobilization in polysaccharide carriers [24,27], on glass strips [28], in sol-gel-derived silica [29], on nanofiber membranes [30], in metal-organic frameworks [31], on gold [32] and silver [33] nanoparticles, on quantum dots [34,35], and on magnetic nanoparticles [36,37,38]. In a number of studies, enzyme activity was diminished because of conformational changes or light absorption by nanostructures.

Of all immobilization methods, the simplest and cheapest is luciferase entrapment in polysaccharide and protein gels such as starch and gelatin gels [39,40]. Previous research showed that the immobilization of bacterial luciferase in starch gel enhanced the stability of the coupled enzyme system of bioluminescent bacteria [25,41]. Moreover, as various enzymes function in close contact with other components of the cell, including lipids, proteins, and carbohydrates, entrapment of the enzymes in such natural polymers as starch and gelatin not only results in practical advantages but also offers new knowledge of the enzyme *in vivositsit* function [42].

The present study describes a simple and relatively cheap method of producing a reagent based on the firefly luciferin-luciferase system immobilized in gelatin gel, which is intended for detecting microbial contamination.

## 2. Materials and Methods

### 2.1. Reagents

Mutant firefly luciferase (FLuc: EC 1.13.12.7) from *Luciola mingrelica* was used in this study. One vial contained 40 µL of the enzyme solution of concentration 27.2 mg·mL^−1^ and activity 5.5 × 10^12^ (RU·mg^−1^). For analysis, 5 µL of the enzyme solution was dissolved in 125 µL of Tris-acetate buffer, pH 7.8, and 4 µL of the resulting enzyme solution was added to the reaction mixture.

D-luciferin was purchased from Lumtek (Moscow, Russia). EDTA and magnesium sulfate were the products of Medigen (Novosibirsk, Russia), while Tris, bovine serum albumin (BSA), dithiothreitol (DTT), and adenosine-5′-triphosphate (ATP) were purchased from Sigma-Aldrich (St. Louis, MO, USA). Gelatin was procured from Fluka (Buchs, Switzerland), and sucrose was purchased from Gerbu (Heidelberg, Germany). *Escherichia coli* (*E. coli*) cells (BL21 codon Plus (DE3) RIPL) were used as a standard bacterial strain for real sample analysis. Bacterial cell lysis was carried out using a UZDN-2T ultrasonicator from Ukrrospribor (Sumy, Ukraine).

A stock solution of D-luciferin was prepared in Tris buffer containing 0.1 M Tris, 2 mM EDTA, 10 mM MgSO_4_, and glacial acetic acid to lower the pH to 7.8.

Stock solutions of 0.5 mM ATP were prepared using distilled water, 5 mM DTT, 12.5 mg·mL^−1^ BSA, and 0.3 M sucrose in Tris buffer pH 7.8. The working solutions were prepared by further dilution of the stock solutions.

### 2.2. ATP Measurement Using Firefly Luminescent System

The relationship between the intensity of light emission by the luminescent system and the ATP level was determined as follows. We prepared the stock solution of the reaction mixture (290 µL of the buffer, 20 µL of luciferase, and 20 µL of luciferin of various concentrations per measurement). The stock solution was incubated in a thermostatically controlled cabinet at 24 °C for 1 h; then, 330 µL of the stock solution was transferred to the bioluminometer cuvette. The cuvette was placed into the bioluminometer, and background luminescence was measured. Then, 20 µL of 50 nM ATP solution was added to the cuvette, the solution was mixed, and the maximum luminescence was measured (I). To study the effects of stabilizers—DTT, BSA, and sucrose—on FLuc, the solutions of stabilizers were also added to the stock solution.

### 2.3. Immobilization of Firefly Luciferase and D-Luciferin

Immobilization of FLuc was performed by gel entrapment. Gelatin gel was prepared as follows. A weighted amount of gelatin was mixed with Tris buffer pH 7.8 and kept at room temperature for 30 min. After it swelled, the suspension was heated to 80 °C and cooled to 25 °C. The resultant gelatin gel was mixed with FLuc, sucrose, DTT, and BSA solutions, and 25 μL of the mixture was pipetted on the hydrophobic film. The reagents were dried at 8 °C for 24 h.

The starch gel was prepared as follows. Starch was dissolved in Tris buffer pH 7.8, heated for 2 min, and then cooled to 25 °C; after that, FLuc, sucrose, BSA, and DTT solutions were added, and 25 μL of the mixture was pipetted on the hydrophobic film.

Entrapment of D-luciferin into the gelatin and starch gels was performed using the procedure described for FLuc.

### 2.4. Measuring the Activity of Immobilized Reagents

Luminescence measurements were performed using the immobilized FLuc in the presence of ATP (ATP range from 0.3 pM to 3 nM). The calibration curve for standard ATP was obtained by dispensing the reactants into a luminometric tube in the following sequence: one disc of immobilized FLuc; 310 μL of Tris buffer pH 7.8; 20 μL of D-luciferin solution. The reaction mixture was evenly mixed in the tube and kept for 1 min at room temperature; after that, the luminescence baseline was recorded using a luminometer. 20 μL of standard ATP was injected into the tube. The maximum luminescence intensity (I) was measured in relative luminescence units (RLU), and a calibration curve was plotted. The same assay procedure was employed to determine the number of *E. coli* cells, where 20 μL of cell suspension was used instead of ATP solution.

When the luminescence intensity was measured in the presence of immobilized D-luciferin, one disc of immobilized D-luciferin was placed into a luminometric tube, then 4 μL of luciferase solution and 310 μL of Tris buffer were added into the tube, and the reaction mixture was incubated at room temperature. After 60 min, 20 μL of standard ATP solutions were injected to measure the maximum luminescence intensity. In the control measurement, the reaction mixture contained 4 μL of FLuc solution and 20 μL of D-luciferin solution.

The luminescence intensity was measured in the presence of two immobilized reagents containing FLuc and D-luciferin separately. One immobilized FLuc reagent and one immobilized D-luciferin reagent were placed into a luminometric tube, 310 μL of Tris buffer pH 7.8 was added, and the reaction mixture was kept at room temperature. After 60 min, the baseline was recorded, and 20 μL of standard ATP solutions were injected to measure the maximum luminescence intensity.

Luminescence measurements were performed using a Lumat LB 9507 luminometer, Berthold Technologies, Bad Wildbad, Germany.

### 2.5. Biomass Cultivation

A loopful of freshly cultured *E. coli* cells was taken from a slant and transferred to 50 mL sterile Luria Bertini (LB) broth in an Erlenmeyer flask; the cells were cultivated at 30 °C under shaking conditions for 18 h in an incubator shaker (New Brunswick Excella E25, Eppendorf, Germany). The approximate number of *E. coli* cells in the culture broth was measured using a UV-Vis spectrophotometer UVIKON 943 (Kontron Instruments, Milano, Italy) at 600 nm (1 unit of optical density corresponds to 5 × 10^8^ cells·mL^−1^).

## 3. Results

### 3.1. Determining the Proportions of Components in the Firefly Bioluminescent System

**Figure 1:**
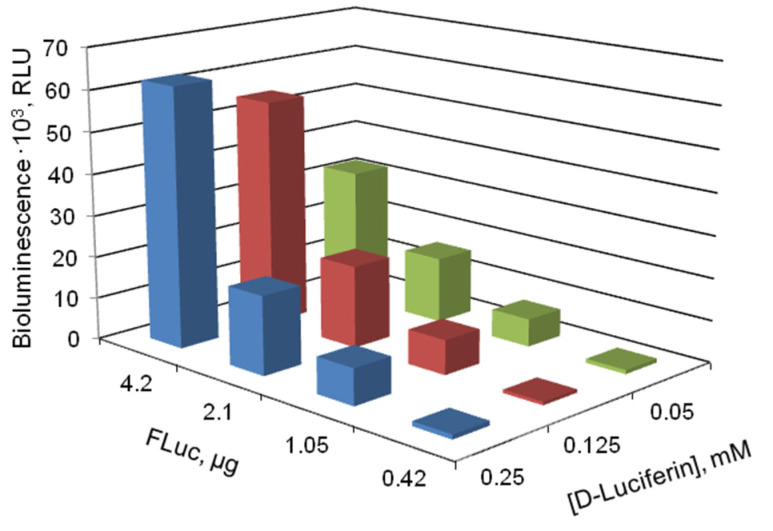
Luminescence intensity of the firefly bioluminescent system vs. the amounts of luciferase (FLuc) and D-luciferin in the reaction mixture. FLuc and D-luciferin were added to the reaction mixture in the form of solutions.

The sensitivity of the immobilized reagent to ATP is evidently determined by the proportions of components in the reagent. Therefore, first, we added different amounts of the components of the enzymatic reaction (FLuc and D-luciferin) to the reaction mixture to determine the proportion that would provide the highest sensitivity of the firefly bioluminescent system to ATP. In those preliminary experiments, the enzyme and the substrate were used in the form of solutions. Figure 1 shows the luminescence intensity of the firefly luciferin-luciferase system as dependent on the amount of FLuc and concentration of D-luciferin in the reaction mixture.

Luminescence intensity reached its maximum at 4.2 µg FLuc and 0.125 or 0.25 mM D-luciferin in the reaction mixture. It is also important that the ratio of the luminescence in the presence of ATP to the background luminescence was the highest under the same conditions.

### 3.2. Choosing the Stabilizer to Be Assessed to The Immobilized Reagent

Additional stabilizers—BSA, DTT, and sucrose—were used to enhance the stability and activity of immobilized reagents and increase their storage time.

Different concentrations of the stabilizers were added to the reaction mixture containing FLuc, D-luciferin, and Tris-acetate buffer pH 7.8, and the mixtures were incubated at 24 °C for 1 h.

**Figure 2:**
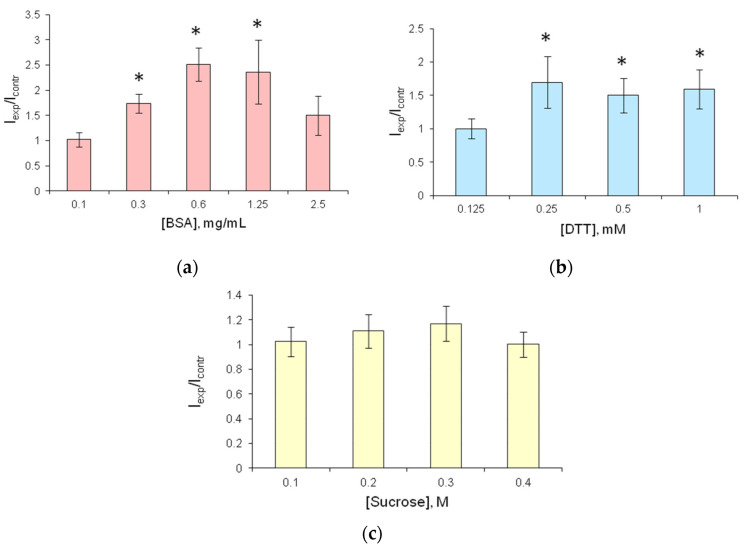
Relative luminescence intensity of soluble FLuc as dependent on concentrations of stabilizers in the reaction mixture after incubation at 24 °C for 1 h. The reaction mixture contained 4.2 µg FLuc, 0.125 mM D-luciferin, and different concentrations of stabilizers: (**a**) BSA, (**b**) DTT, (**c**) sucrose. I_contr_—luminescence intensity of the reagent with no stabilizer added; I_exp_—luminescence intensity of the reagent with a stabilizer. The bioluminescent reaction was started by adding 20 µL of 50 nM ATP to the reaction mixture. * *p* < 0.05 when comparing with the control.

The addition of BSA to the reaction mixture resulted in an increase in bioluminescence intensity by a factor of 2.5 relative to the control (Figure 2a). The highest luminescence intensity was recorded at BSA concentration of 0.6 and 1.25 mg·mL^−1^.

The addition of DTT at a concentration of 0.25 mM or higher increased the luminescence intensity of the bioluminescent system by a factor of 1.5 (Figure 2b). In the presence of sucrose, however, no increase in the enzyme activity was observed (Figure 2c).

### 3.3. Activity of Immobilized FLuc in The Presence of Stabilizers

**Figure 3:**
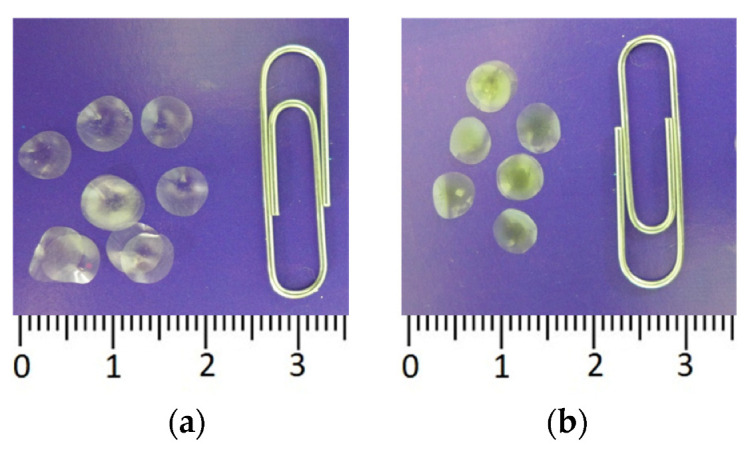
Immobilized reagents based on firefly luciferin-luciferase system: (**a**) Reagent containing 4.2 µg FLuc immobilized in 1% gelatin gel; (**b**) Reagent containing 0.125 mM D-luciferin in 1% gelatin gel.

Experiments were carried out to study the possibility of entrapping firefly luciferase in the starch and gelatin gels with different percentages of starch (3, 4, and 5%) and gelatin (1, 1.2, 1.5, and 1.7%). Those percentages of starch and gelatin were chosen because the resulting gels were conveniently measured and dispensed using a pipette and because those gels were capable of forming thin strong discs after drying, which is important for producing single-use reagents for assays. To immobilize the enzyme, 25 µL of each gel suspension containing FLuc was pipetted onto the fluoropolymer film and dried. The greatest activity of immobilized FLuc was achieved with 1% gelatin gel. The resultant reagents were in the form of thin discs 6–7 mm in diameter (Figure 3a). We failed to separate the dry starch gel-based discs from the hydrophobic film onto which the starch suspension had been pipetted: the discs crumbled and, thus, were unusable.

To produce the enzyme preparation with good storage stability, the gelatin gel used for the entrapment of FLuc was supplemented with stabilizers: sucrose, DTT, and BSA at different concentrations.

**Figure 4:**
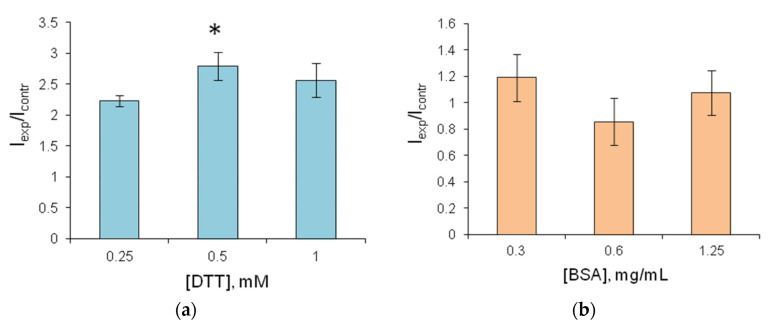
Relative luminescence intensity of FLuc entrapped in 1% gelatin gel with stabilizers. (**a**) The reagent contains various concentrations of DTT; I_contr_—reagent luminescence intensity without DTT; I_exp_—reagent luminescence intensity in the presence of DTT; (**b**) The reagent contains 0.5 mM DTT and, additionally, BSA at various concentrations; I_contr_—reagent luminescence intensity in the presence of 0.5 mM DTT, with no BSA; I_exp_—reagent luminescence intensity in the presence of DTT and BSA. * *p* < 0.05 when compared with value in the presence of 0.25 mM DTT.

**Figure 5:**
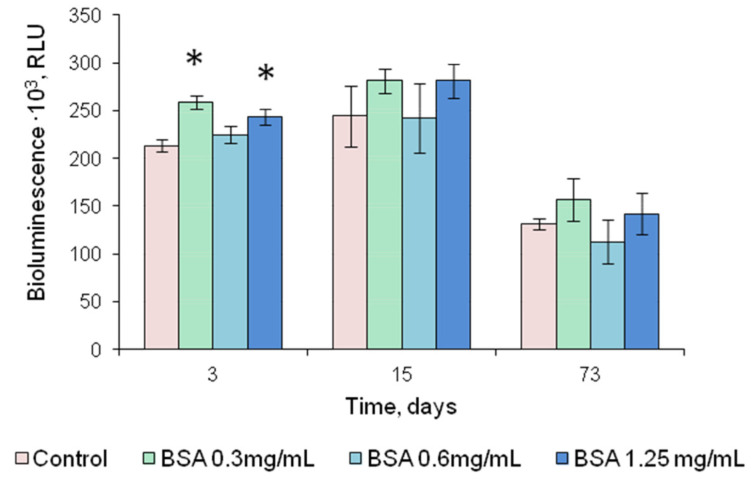
Luminescence intensity of FLuc entrapped in 1% gelatin gel in the presence of the stabilizers, DTT and BSA, as dependent on the period of storage at 4 °C; DTT concentration in the reagent is 0.5 mM. Control—luminescence intensity of FLuc entrapped in 1% gelatin gel in the presence of 0.5 mM DTT. * *p* < 0.05 when comparing with the control.

The addition of sucrose to the gelatin gel used for FLuc entrapment prevented the reagents from drying up, and they were destroyed when separated from the hydrophobic film. Therefore, sucrose was considered unsuitable for use as a stabilizer. The addition of DTT increased FLuc luminescence intensity by a factor of 2–2.5 (Figure 4a). After 2.5-month storage, the reagent containing FLuc immobilized along with 0.5 mM DTT retained 60% of its initial activity (Figure 5). Results of the experiments with BSA were contradictory. Although BSA was a factor in stabilizing soluble FLuc (Figure 2a), and although another study showed that the firefly luciferase stability was enhanced and the storage time of the reagent was increased in the presence of BSA [43], in the present study, the addition of BSA to the immobilized reagent containing 0.5 mM DTT did not lead to higher activity of immobilized FLuc (Figure 4b). Moreover, the addition of BSA did not increase the reagent storage period (Figure 5). The activity of FLuc immobilized in the presence of 0.5 mM DTT (control) was almost the same as the activity of FLuc immobilized in the presence of both 0.5 mM DTT and BSA at various concentrations.

### 3.4. Analytical Characterization of Immobilized FLuc

**Figure 6:**
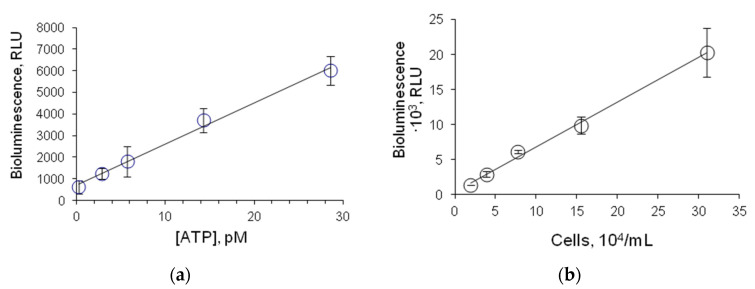
Luminescence intensity of FLuc entrapped in 1% gelatin gel: (**a**) as dependent on ATP concentration in the solution; (**b**) as dependent on the number of *E. coli* lysed cells in the reaction mixture. One disc of immobilized reagent contained 4.2 µg FLuc.

FLuc-based assessment of microbial contamination of various media is conducted by measuring ATP in the solution containing lysed bacterial cells. Therefore, we plotted a calibration curve showing ATP concentration dependence of the activity of FLuc entrapped in 1% gelatin gel. The minimum detectable concentration of ATP was 0.3 pM (Figure 6a), and the linear response range was between 0.3 pM and 3 nM.

*E. coli* cells were used as a microbial sample—an ATP source. A correlation was found between the luminescence intensity of FLuc entrapped in 1% gelatin gel and the number of *E. coli* lysed cells in the reaction mixture (Figure 6b). The limit of detection was defined as the concentration of cells in the reaction mixture at which FLuc luminescence intensity was higher than the background luminescence intensity by a factor of 3. The limit of detection was determined as 20,000 cells·mL^−1^.

### 3.5. D-Luciferin Immobilization

In order to simplify the process of analyzing microbial contamination using the firefly luciferin-luciferase system, we tested the possibility of immobilizing firefly D-luciferin in starch and gelatin gels. D-luciferin immobilization was carried out following the procedure described in Section 2. Like immobilized FLuc, immobilized D-luciferin was in the form of small dry discs (3b), each disc intended for one assay.

**Figure 7:**
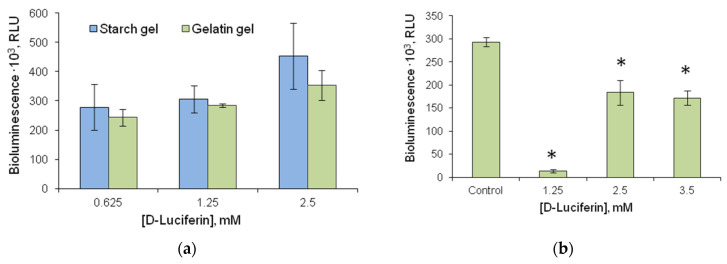
Luminescence intensity of reagents of different compositions based on the firefly luciferin-luciferase system: (**a**) Luminescence intensity of FLuc in the presence of immobilized D-luciferin (FLuc was added to the reaction mixture in the form of the solution; reaction was started by adding 20 µL of 50 nM ATP solution); 3% starch gel and 1% gelatin gel were used for immobilization; (**b**) Luminescence intensity of multicomponent reagent containing 4.2 µg of FLuc and various concentrations of D-luciferin; reaction was started by adding 20 µL of 50 nM ATP solution; 1% gelatin gel was used for immobilization. * *p* < 0.05 when comparing with the control.

Luminescence of the firefly luciferin-luciferase system insignificantly increased with an increase in D-luciferin concentration in the immobilized reagent (Figure 7a). No significant differences were observed in the activity of the bioluminescent system whichever gel type was used to immobilize D-luciferin. However, in the experiment with D-luciferin immobilized in a starch gel, bioluminescence signal intensity varied considerably, leading to poor reproducibility of results. Thus, gelatin gel was the better carrier for D-luciferin, with the ATP limit of detection being 0.3 pM.

The process of analyzing ATP levels using the firefly luciferin-luciferase system can be simplified further by co-immobilizing D-luciferin and FLuc. In this study, we prepared reagents containing FLuc and its substrate, D-luciferin, co-immobilized into 1% gelatin gel. The activity of multicomponent reagents was determined by the concentration of the added D-luciferin (Figure 7b). The optimal D-luciferin concentration was 2.5 mM, and the maximum luminescence intensity of the reagent containing that concentration of D-luciferin was 40% lower than that of FLuc immobilized with no D-luciferin (control). Thus, the co-immobilization of FLuc and D-luciferin led to a decrease in the activity of the immobilized reagent. Nevertheless, the remaining activity was sufficiently high for the multicomponent reagent to be used in microbial contamination assay, as the limit of detection of ATP using the multicomponent reagent was 0.6 pM.

**Figure 8:**
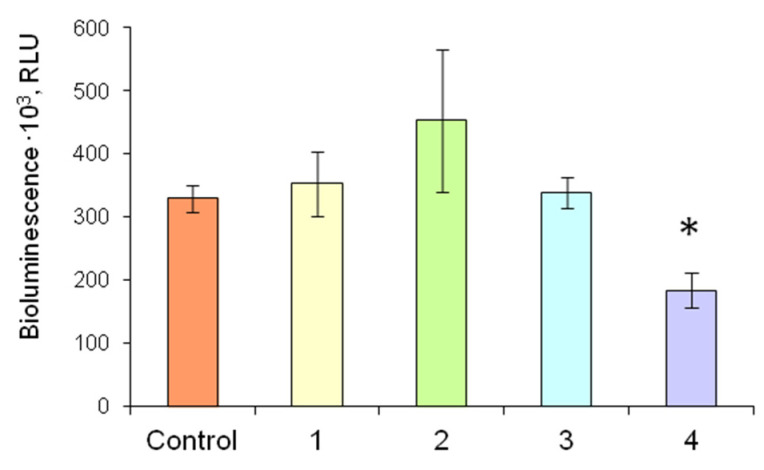
Luminescence intensity of the firefly luciferin-luciferase system in reaction mixtures of different compositions in the presence of 3 nM ATP: Control—Reaction mixture contained solutions of 4.2 µg of FLuc and 2.5 mM D-luciferin; 1—Reaction mixture contained a solution of 4.2 µg of FLuc and 2.5 mM D-luciferin immobilized in gelatin gel; 2—Reaction mixture contained a solution of 4.2 µg of FLuc and 2.5 mM D-luciferin immobilized in starch gel; 3—Two different immobilized reagents based on gelatin gel—a disc with immobilized FLuc (4.2 µg of FLuc in each disc) and a disc with immobilized D-luciferin (D-luciferin concentration was 2.5 mM)—were added to the reaction mixture; 4—The multicomponent reagent containing 4.2 µg of FLuc and 2.5 mM D-luciferin co-immobilized in gelatin gel was added to the reaction mixture. * *p* < 0.05 compared with the control.

Figure 8 demonstrates the comparison of different levels of firefly bioluminescent system activity as dependent on the conditions of the analysis. In the experiments with FLuc and D-luciferin immobilized in 1% gelatin gel separately and jointly, luminescence intensity was similar to that in the control, i.e., the luminescence intensity of soluble FLuc in the presence of D-luciferin solution.

## 4. Discussion

Although the firefly luciferin-luciferase system has been fairly commonly used in different branches of biotechnology, medicine, and the food industry, etc., it still attracts lively interest. Considerable research is undertaken to find ways to stabilize FLuc in order to improve its performance and keep it active during storage. One such way is to immobilize FLuc. In the current study, we investigated the possibility of immobilizing firefly luciferase in starch and gelatin gels as the basis for constructing a pre-dosed reagent for assessing microbial contamination. We use mutant firefly luciferase from *Luciola mingrelica* characterize by high stability and relatively low cost [44].

Although starch gel was previously used to effectively immobilize other enzyme systems such as the bioluminescent enzyme system of luminous bacteria [39,45] and butyrylcholinesterase [46,47,48], this carrier proved unsuitable for immobilizing firefly luciferase. Components of buffer solutions seemed to interact with starch gel, preventing complete drying of the discs with immobilized FLuc. Therefore, it was impossible to separate the discs from the hydrophobic film, and storing and using them was problematic.

Gelatin gel was chosen for immobilizing FLuc not only because of its relatively low cost or simple immobilization procedure but also because a previous study showed that no substantial changes occurred in the function of the firefly luciferin-luciferase system in 5% gelatin gel [40]. In the present study, we used 1% gelatin gel, whose consistency enabled pipetting the prepared mixture onto hydrophobic film in order to produce single-use reagents for analyzing microbial contamination based on the measured ATP levels.

To maintain the activity of immobilized FLuc, we used stabilizers, which differed in their mechanisms of action on enzymes. Three types of stabilizers are generally used to maintain the catalytic activity of enzymes: (1) stabilizers regulating the hydration shell of the enzyme (osmolytes); (2) stabilizers of the enzyme SH-groups, such as DTT and mercaptoethanol; (3) stabilizers enhancing solution viscosity by increasing total protein concentration, e.g., albumins.

Osmolytes as stabilizers are basically divided into three classes: polyols and sugars (sorbitol, glycerol, sucrose, and trehalose), amino acids and their derivatives (glycine, taurine, proline, and betaine), and methyl amines (TMAO, choline-o-sulfate, and sarcosine) [49,50]. Compounds of each class interact with both the peptide backbone and amino acid residues of the side chains. Osmolytes alter the functions of proteins by changing their stability under physiological conditions through increasing melting temperature and the Gibbs free energy, which is responsible for the balance between the native and denatured states [51]. One of the commonly used osmolytes is sucrose. It is widely used in vitro as an additive to protein formulations to protect labile proteins from the harmful effects of high temperature, freezing, and drying [52]. Luciferase is an unstable enzyme, losing its activity rather quickly, especially at high temperatures. However, a number of authors reported that sucrose and trehalose influence the stability, kinetic properties, and structure of the enzyme [53,54]. The enzyme activity is increased in the presence of sucrose and trehalose at high temperatures and the optimum temperature varies from 25 to 30 °C, which may be associated with the increase in the surface tension and protein rigidity. The presence of molar concentrations of sugars increases protein rigidity due to the higher viscosity of the medium. Moreover, the addition of stabilizers decreases the light decay rate, which is associated with aggregation inhibition—one of the main mechanisms of irreversible thermoinactivation [53]. Although sucrose stabilizes FLuc, preventing its conformational changes and thermoinactivation, in the present study, we elected not to use it, as the addition of sucrose as a stabilizer prevented complete drying of the reagents, which were, thus, damaged and inconvenient to use.

To “protect” the protein SH-group from uncontrolled oxidation, DTT and mercaptoethanol are used. DTT prevents the formation of incorrect intramolecular and intermolecular -S-S- bonds, which lead to the formation of dimers and polymers, whose biological activity is considerably lower than that of monomer molecules. Mercaptoethanol is an analog of DTT, which is used to protect sulfhydryl groups of protein molecules (including enzymes) from oxidation and, otherwise, to protect readily oxidized compounds from the impact of oxygen. These compounds were effective stabilizers of the bioluminescent enzyme system of luminous bacteria [25]. Luciferase of firefly *Luciola mingrelica* contains eight cysteine residues, two of which are located on the surface of the protein globule. Oxidation of SH-groups of these cysteines results in enzyme inactivation. The addition of DTT to the enzyme preparation was found to stabilize the luciferase of firefly *Luciola mingrelica* and to decrease the rate constant of thermal inactivation by a factor of three [55]. In the current study, we also observed an increase in luminescence intensity of immobilized FLuc by a factor of 2–2.5 when DTT was added to the gelatin gel used as a carrier for immobilization.

Among albumins, BSA is the most frequently used stabilizer [56]. The stabilizing effect of albumins was associated with both an increase in the total viscosity of protein solutions and the ability of albumins to bind substrates and/or products of enzymatic reactions. For instance, BSA bound long-chain-length aldehyde (substrate of bioluminescent reaction of luminous bacteria), preventing bioluminescence inhibition by high aldehyde concentrations [57].

The effects of the substances influencing enzyme activity, when they are present together under certain conditions, may be nonadditive. A study by Tomoyasu et al. [58] showed that at a temperature of 42 °C, the simultaneous addition of DTT and BSA caused a reduction in the stabilizing effect on the activity of *Renilla* luciferase compared to the stabilization resulting from the addition of BSA alone. The authors think that under elevated temperature, 17 disulfide bonds in the native BSA molecule, stabilizing the protein tertiary structure, are broken, and, in the presence of DTT, the denatured BSA molecules are aggregated. At the same time, the authors note that at lower temperatures (30 °C), BSA does produce a stabilizing effect in the presence of DTT. The stabilizing effect of BSA on the luciferase of firefly *Photinus pyralis* was retained in the presence of 1 mM DTT even after 4 days of incubation [58]. In the present study, although the addition of BSA had a stabilizing effect on soluble FLuc and increased its activity (Figure 2a), the addition of BSA to the immobilized reagent neither substantially enhanced the activity of immobilized FLuc nor resulted in a considerable stabilizing effect during storage. Nevertheless, as the reagents are intended for use not only in the laboratory but also in the field, we simultaneously added 0.3 mg·mL^−1^ BSA and 0.5 mM DTT to the reagents.

The use of immobilized FLuc as a single-use pre-dosed reagent simplifies the assay procedure. Yet, the necessity to dose the D-luciferin solution remains a disadvantage. To simplify the analysis, even more, different components of enzyme systems can be co-immobilized. A similar approach was successfully used previously, during the development of a number of multicomponent enzyme preparations. Several studies [45,59,60,61] described the production of multicomponent reagents containing a bioluminescent coupled enzyme system of luminous bacteria and its substrates—NADH and myristyl aldehyde. Butyrylcholinesterase was incorporated into the immobilized preparation simultaneously with the indicator of thiol groups—5-5′-dithiobis (2-nitrobenzoic acid) [48]. Co-immobilization of FLuc and D-luciferin was also performed: a study by Nguyen et al. [62] described a method of producing a reagent for assessing microbial contamination of the air based on ATP levels, where FLuc and D-luciferin were co-immobilized on paper discs, and the limit of detection of cells was 2.32 × 10^3^ cells·mL^−1^. Another study described a biosensor based on FLuc and D-luciferin immobilized on paper discs, with a sensitivity of 10 fmol ATP [63].

In the current study, immobilization of D-luciferin separately or together with FLuc considerably simplified the process of ATP quantification in the sample. When FLuc and D-luciferin were immobilized separately, to determine ATP, two immobilized reagents, one containing the enzyme (FLuc) and the other substrate (D-luciferin), were placed into the cuvette with the sample (Figure 3). When multicomponent reagent was used, one disc, containing co-immobilized FLuc and D-luciferin, was placed into the tested solution. In the first case, the sensitivity of reagents to ATP was 0.3 pM, and in the second case, the limit of detection of ATP was lower by a factor of 2–0.6 pM. Thus, we developed a number of reagents that can be quickly and conveniently used for in situ assays and obviated the need for the enzyme and substrate in the form of solutions.

## 5. Conclusions

The present study demonstrated that FLuc immobilized into gelatin gel together with the stabilizers DTT and BSA showed rather high sensitivity to ATP and bacterial cells. Moreover, we prepared reagents containing D-luciferin immobilized separately or together with FLuc. Hence, reagent solutions need not be used in ATP determination, which considerably simplifies the procedure of the analysis. Thus, we developed a number of single-use pre-dosed reagents that can serve as the basis for optical biosensors to analyze microbial contamination of various media.

## 6. Patents

Patent RF N 2654672. Complex of reagents for the adenosine-5′-triphosphate quantitative analysis. Kirillova M.A., Esimbekova E.N., Kratasyuk V.A.

## Data Availability

Not applicable.
